# Identification of sources of resistance to scald (*Rhynchosporium commune*) and of related genomic regions using genome-wide association in a mapping panel of spring barley

**DOI:** 10.3389/fpls.2023.1133404

**Published:** 2023-11-28

**Authors:** Houda Hiddar, Sajid Rehman, Bouchra Belkadi, Abdelkarim Filali-Maltouf, Muamar Al-Jaboobi, Ramesh Pal Singh Verma, Sanjaya Gyawali, Zakaria Kehel, Ahmed Amri

**Affiliations:** ^1^ Laboratory of Microbiology and Molecular Biology, Faculty of Sciences, BioBio Research Center, University Mohammed V in Rabat, Rabat, Morocco; ^2^ Biodiversity and Crop Improvement Program, International Center for Agricultural Research in the Dry Areas (ICARDA), Rabat, Morocco; ^3^ Olds College, Field Crop Development Center, Lacombe, AB, Canada; ^4^ Indian Institute of Wheat and Barley Research (IIWBR), Karnal, Haryana, India; ^5^ Mount Vernon North-Western Washington Research & Extension Center, Washington State University, Mount Vernon, WA, United States

**Keywords:** barley, *Rhynchosporium commune*, resistance, SNP, GWAS, quantitative trait loci

## Abstract

Barley is an important crop worldwide known for its adaptation to harsh environments and used in multiple forms as feed, food and beverages. Its productivity is affected by major abiotic and biotic stresses. Scald caused by hemibiotrophic fungus *Rhynchosporium commune* is a major foliar disease in many parts of the world. Host plant resistance is targeted by breeders to efficiently control this disease. An association mapping panel of 316 spring barley genotypes (AM2017) was screened for seedling resistance in greenhouse against three *R. commune* isolates and for adult plant resistance in three field locations in Morocco. The phenotyping results showed different numbers of entries with resistant and moderately resistant reactions at both seedling and adult plant stages. The reactions differed between the isolates with the highest percentage of resistant genotypes observed for isolate SC-S611 (49.4%) and highest percentage of susceptible genotypes (73.8%) for isolate SC-1122. At adult plant stage, the highest percentage of scald resistant genotypes (64.5%) was observed at Rommani site compared to 56% at Guich site and only 28.8% at Marchouch site. Seven genotypes were resistant at the seedling and adult plant stages. Genome wide association study (GWAS) revealed 102 MTA (15 QTL) at the seedling stage, and 25 MTA (12 QTL) associated with scald resistance at the adult plant stage. In addition, the sequences of 92 out of 102 at SRT, and 24 out of 25 significant SNP markers at APR were located in genomic regions enriched with functional proteins involved in diverse cellular processes including disease resistance. These markers span over all chromosomes with the majority of SNPs located on 3H and 7H. This study has verified 18 QTL reported in previous studies. In addition, it was successful in identifying new sources of resistance and novel genomic regions which could help in enhancing scald resistance in barley breeding programs.

## 1 Introduction

Barley ranks the second most important temperate cereal crop in the world after *Triticum aestivum L*. Globally, it was sown over 51 million hectares with a production of 150 million metric tons in 2019 (FAOSTAT, 2020). Barley use is not limited only to feed, food and beverages, but several studies have highlighted it’s nutritional value and health benefits including the potential for reducing colon cancer incidence, and lowering cholesterol (FDA, 2006; Kawka et al., 2019; Li et al., 2021).

The quality and productivity of barley crop are affected by abiotic and biotic stress factors. Among biotic stresses, leaf blotch or scald caused by the hemibiotrophic ascomycete fungus *Rhynchosporium commune* (formerly known as *R. secalis*) is one of the most economically important and destructive disease of barley worldwide, which can reduce grain quality and inflict high yield losses (Xi et al., 2000; Perfect and Green, 2001; Zhan et al., 2008). *Rhynchosporium commune* is known for its high genetic diversity which allow the pathogen population to overcome resistant cultivars, and to develop resistance to fungicides within a short period of time (Stefansson et al., 2012; Stefansson et al., 2013; Mcdonald, 2015; Brunner et al., 2016; Mohd-Assaad et al., 2016). The inherent complexity of the fungus requires an integrated disease management approach (Stefansson et al., 2012; McLean and Hollaway, 2018), and genetic resistance is the most economical, sustainable, and environment friendly strategy for controlling barley scald disease.

Development and deployment of resistant barley cultivars through incorporation and pyramiding of both major and minor genes can provide durable resistance against *R. commune* (Walters et al., 2012). Scald disease resistance can be classified as horizontal or vertical, both types of resistance are based on the number of “*R* genes” involved. Vertical resistance is qualitative in nature while horizontal resistance is governed by minor genes.

However, both vertical and horizontal resistance mechanisms are used by breeders. Compared to other barley diseases, relatively a few resistance loci against *R. commune* have been discovered which can be employed in breeding programs (Dracatos et al., 2019; Clare et al., 2020; Zhang et al., 2020). The qualitative resistance genes, frequently detected using specific strains at the seedling stage, provide a high level of resistance at all growth stages (Zhan et al., 2008). However, quantitative resistance *to R. commune* identified at the adult plant stage provide a partial level of resistance (Wallwork and Grcic, 2011; Zhan et al., 2008). The first *R. commune* resistance gene was designated as *Rrs1* which conferred resistance under field conditions, and under controlled conditions with specific isolates (Zhan et al., 2008; Looseley et al., 2018). Frequently, barley scald disease resistance loci were detected on chromosome 3H flanking *Rrs1* locus, and on chromosome 7H flanking *Rrs2* locus (Zhan et al., 2008; Looseley et al., 2018). Across the barley genome, several major and minor *R* genes, and QTLs for leaf scald resistance have been identified in different barley genotypes scattered across the seven barley chromosomes (Zhang et al., 2020). To date, 11 major loci including *Rrs1*, *Rrs2*, *Rrs4*, *Rrs12*, *Rrs13*, *Rrs14*, *Rrs15*, *Rrs16*, *Rrs17*, (*Rrs15* (CI8288), and *Rrs18* controlling resistance to *R. commune* have been identified. Further, the major scald resistance genes have been detected on all chromosomes except for 5H, with *Rrs14* on chromosome 1H, *Rrs17* on chromosome 2H, *Rrs1* and *Rrs4* on chromosome 3H, *Rrs3* and *Rrs16* on chromosome 4H, *Rrs13* and *Rrs18* on chromosome 6H, and *Rrs2*, *Rrs12* and *Rrs15* on chromosome 7H (Bjørnstad et al., 2002; Zhang et al., 2020). Most of the modern barley cultivars share the same allele combinations of resistant loci indicating the limited genetic diversity among the barley germplasm (Williams et al., 2003). Furthermore, owing to the complex virulence spectrum and ability of *R. commune* populations to overcome resistant cultivars, durable resistance against scald disease requires continuous research for polygenic resistance (Xi et al., 2000).

Breeders and researchers are using landraces and wild relatives, genebank accessions, panels and genetic stocks to identify new sources of resistance. There are various sources of resistance derived from *Hordeum vulgare* ssp*. vulgare* (Silvar et al., 2010; Hofmann et al., 2013), from wild barley *Hordeum vulgare* ssp*. spontaneum* (von Korff et al., 2005; Yun et al., 2005) and from *Hordeum bulbosum* (Pickering et al., 2006).

In recent years, with the availability of a high-quality reference genome assembly (Mascher et al., 2017), and of high-density genotyping platforms (Moragues et al., 2010; Comadran et al., 2012), there has been a growing interest in genomic resources of barley. Different statistical methods have been developed and used to identify molecular markers that are linked to traits of interest in bi-parental QTL mapping (linkage mapping) and in genome wide association mapping (Linkage dis-equilibrium mapping). The allelic richness and the genetic mapping resolution in bi-parental mapping populations are mainly affected by the genetic recombination and segregation (Alqudah et al., 2020). Genome-wide association studies (GWAS) have proven to be an efficient tool that captures greater genetic variation and offers high resolution mapping of QTL and associated candidate genes compared to classical linkage analysis (Cockram et al., 2008; Comadran et al., 2012; Bayer et al., 2017). In barley, GWAS has been successfully applied to identify QTLs associated with resistance to net blotch (Amezrou et al., 2018), powdery mildew (Czembor et al., 2022), spot blotch (Visioni et al., 2020), and barley stripe rust (Visioni et al., 2018).

The main objectives of this study were to identify sources of resistance to scald in a diverse panel of barley genotypes at the seedling and at the adult plant stages, and to use GWAS to identify the underlying genomic regions conditioning resistance to *R. commune*.

## 2 Materials and methods

### 2.1 Plant material

A collection of 316 spring barley genotypes designated as association mapping-panel 2017 (AM2017) constructed by barley breeders at the International Center for Agriculture Research in the Dry Areas (ICARDA) was used in this study. It has 143 six-row and 173 two-row type barley genotypes, consisting of 134 advanced breeding lines from ICARDA, 21 landraces from ICARDA genebank, 161 released cultivars from Asia, Africa, Europe, and America (Verma et al., 2021).

### 2.2 Seedling stage disease resistance test

Scald infected barley leaves were collected from naturally infected barley fields from different agro-ecological zones of Morocco during the disease surveys conducted from 2015 to 2018. Isolation of *R. commune* was performed by soaking the scald infected barley leaves in sterile distilled water for 15 min followed by surface sterilization using 10% sodium hypochlorite solution for 30 s and 50% ethanol for 15 s, rinsed with sterile distilled water three times, and dried up within two layers of sterile Whatman filter paper. The leaf segments were incubated at 14°C in darkness for two weeks on Lima Bean agar (LMA) supplemented with Kanamycin and Streptomycin (50 mg per liter). After two weeks of incubation, the sporulating white or pinkish colonies were streaked on new LBA plates for the preparation of single conidial isolates.

Four seeds of each genotype were planted in a single cone of 3.8 cm diameter and 14 cm depth containing peat moss supplemented with 14-14-14 NPK fertilizer in 98-Ray Leach containers (Stuewe and Sons, Inc., Oregon, USA) in 3 replications. Seedlings of the AM2017 were grown under controlled conditions with a photoperiod of 16 h light/8 h dark at 20 ± 1 ° C in the growth chamber (Model MC1750; Snijder Scientific, Tilberg, Netherlands) at ICARDA, Rabat, Morocco. Three virulent isolates of *R.* commune, SC-511, SC-1122 and SC-611 collected from Marchouch (MCH), Sidi Allal Tazi (SAT), and Annoceur (ACR) were used for seedling resistance. The inoculum was prepared from the 10-12 days old LBA plates by rubbing the agar surface with sterile glass slide followed by filtration through double layer of cheese cloth. The inoculation of about 2 weeks old seedlings, with the second leaf fully extended, was performed using spore suspension of 5×10^5^ conidia/ml supplemented with surfactant Tween 20 (0.01%) followed by incubation at 100% relative humidity in dark for 72 h at 15°C in the growth chamber. Then the seedlings were transferred to a greenhouse with day/night temperature regime of 20°C/16°C with a photoperiod of 16h light and 8h of darkness. After 16 days post infection (dpi), the infection types were recorded using disease rating scale of 0 to 5 (Salamati and Tronsmo, 1997) where 0 = I (Immune), 1= R (resistant), 2 = MR (moderately resistant), 3 = MS (moderately susceptible), 4 = S (susceptible), and 5 = HS (highly susceptible).

We also checked the virulence spectrum of three *Rhynchosporium commune* (*Rc*) isolates SC-1122, SC-511, and SC-611 on nine barley differential Atlas, Atlas 46, Brier, CI3515, CI4364, Jet, La Masita, Osiris, and Turk. These differentials possess specific resistance genes (*R*-genes) and have been previously characterized for their response to *Rc* isolates. In addition, barley differential interactions were examined to determine the compatibility between the *Rc* isolates and the different host genotypes. The presence or absence of compatible interactions, indicated by disease development, and incompatible interactions, indicated by the absence of disease symptoms, were recorded for each combination.

### 2.3 Adult plant stage disease assessment

Field trials were conducted in Morocco at the INRA experimental stations of Marchouch (MCH; 33° 56 N, 6° 63 W) and Guich (33° 58 N, 6° 51 W), and at Rommani region during the cropping season of 2018-2019. Each entry was planted as paired row of 1 meter length with row spacing of 0.5 m in an augmented design using two susceptible (Tissa and Tocada) and two resistant checks (Atlas 46 and ICARDA 4). To ensure high disease pressure, each block was surrounded by a border composed of a mixture of scald susceptible genotypes (Tissa, Aglou, Tiddas, Fleet, Adrar, Shepherd, Baudin and Alester).

Starting from Zadoks scale GS30 (Zadoks and Board, 1974) at an interval of 10-12 days, the trials were artificially inoculated four times using a knapsack sprayer with inoculum composed of a mixture of 15 isolates of *R. commune* which were collected from different agro-ecological zones of Morocco. Furthermore, scald infected barley residues from the previous growing season were uniformly distributed over the trial to ensure disease establishment. In addition, the development and spread of the disease was favoured by applying frequent mist irrigation in late afternoons daily. The disease severity was assessed at GS 73-75 using 0-9 scale (Saari and Prescott, 1975), and the genotypes were categorized into five classes; 0 to 2 as resistant, 3 to 4 as moderately resistant, 5 to 6 as moderately susceptible, 7 to 8 as susceptible and 9 as highly susceptible. The area under the disease progression curve (AUDPC) was calculated using the following equation (Jeger and Viljanen-Rollinson, 2001).


$$\text{AUDPC}=\sum_{a}^{1}i=1^{\text{n}}[({\text{RC}}_{i+1}+{\text{RC}}_{i})/2][({\text{t}}_{i+1}-{\text{t}}_{i})]$$

Where RC*_i_* = *R. commune* severity on *i*th days, t*_i_* = time in days at *i*th observation, and *n* is the total number of observations.

### 2.4 Data analysis

The frequencies of different classes of reaction to *R. commune* at the seedling and adult plant stages were determined using R software (R Core Team, 2019) and the Venn diagrams were developed to show the number of resistant entries common between the three isolates and among the three fields.

To gain a comprehensive understanding of the genetic control of scald resistance and its potential for improvement through breeding efforts, narrow-sense heritability (H^2^) of scald genetic resistance was estimated for each test at the seedling and adult plant stages using the variance component method. The heritability estimate was calculated as the ratio of additive genetic variance to the total phenotypic variance (Falconer and Mackay, 1996) with the use of R software.

### 2.5 Genotyping, population structure, and linkage disequilibrium of AM2017

Genomic DNA extraction, genotyping, population structure, and genetic diversity of the 316 genotypes used in this study has been described in an earlier study by Verma et al. (2021). Isolation of genomic DNA was conducted at the growth stage (GS12) using a lyophilized young leaf tissue from a single plant at the Cereal Crop Research Unit, USDA-ARS, Fargo, North Dakota, USA (Slotta et al., 2008). The Illumina iSelect 50K SNP array for barley (Illumina, San Diego, CA, USA) was used to perform SNP genotyping following the manufacturer’s guidelines (Bayer et al., 2017). Further, 36,793 SNP markers were used for genetic analysis after quality control by discarding all monomorphic markers, markers with missing values of more than 20%, and with minor allele frequency (MAF) of less than 5%.

The population structure of AM2017 was assessed using the filtered SNP markers to estimate individual admixture coefficients and was performed using the sparse Non-negative Matrix Factorization (sNMF) algorithm implemented in the R package *LEA* (Frichot and François, 2015). The cross-entropy criterion was used from sNMF function to evaluate several putative populations (K) ranging from K=2 to K=10 (Alexander and Lange, 2011; Frichot et al., 2014).

For each K we set the number of iterations to 200, the number of runs to 10, alpha to 10, and tolerance to 10^-5^. The genotypes were declared admixed or were assigned to subgroups using 80% membership criterion. The Principal Component Analysis (PCA) was performed with the 36,793 filtered SNP markers using TASSEL version 5.0. Furthermore, the kinship matrix (K) among 316 genotypes was computed using filtered set of SNP markers in TASSEL 5.0 (Bradbury et al., 2007).

The linkage disequilibrium (LD) was determined for all pairs of loci using SNP markers with known positions. The squared allele-frequency correlations (r^2^) were computed in PLINK V1.9 using non-linear regression with a threshold set at 0.2 (Amezrou et al., 2018; Visioni et al., 2018; Visioni et al., 2020; Amouzoune et al., 2022). The extent of genome wide LD decay was visualized by plotting intra-chromosomal r^2^ values against the physical distance in cM (Purcell et al., 2007).

### 2.6 Genome-wide association study

The association mapping was conducted in Tassel (version 5.2.53) software by combining phenotypic and genotypic data (Bradbury et al., 2007; Lipka et al., 2012). Two general linear models (GLM+Q), and (GLM+ PCA), and two mixed linear models, (MLM+Q+K), and (MLM+PCA+K), were tested by taking into account kinship (K matrix), population structure (Q matrix), and principal coordinate analysis (PCA) as covariates to control false positives.

In addition, several GAPIT version 3.0 models (Lipka et al., 2012) were used to validate significant SNP markers/loci associated to scald resistance. In GAPIT3, GWAS was performed using Settlement of MLM Under Progressively Exclusive Relationship (SUPER) (Wang et al., 2014), Multiple-locus MLM (MLMM) (Segura et al., 2012), Fixed and random model Circulating Probability Unification (FarmCPU) (Zhang et al., 2010), and Bayesian-information and Linkage-disequilibrium Iteratively Nested Keyway (BLINK) (Huang et al., 2019).

A GWAS threshold P-value of < 2.0 x 10^-4^ [−log10(P value)< 3.7] was used for declaring significant-marker trait associations as reported by Borrego-Benjumea et al. (2021). This hybrid approach was based on the median of two threshold methods: a stringent method proposed by Wang et al. (2014) determines the significance threshold using the equation α = 1/m, where ‘m’ represents the number of markers. In this case, -log_10_(P-value) < 4.4 was considered significant; and a less stringent approach (Chen et al., 2010) employs the use of bottom 0.1 percentile of P-values distribution [−log10(P value)< 3.0]. It balances the need for stringent control of Type I error (false positives) with the goal of discovering meaningful associations. In addition, we also used LD adjusted Bonferroni correction (Duggal et al., 2008; Visioni et al., 2020) which declared the P-value significance threshold to < 2.0 x 10^-4^ [−log10(P value) < 3.7]. Furthermore, QTL being validated by our study were also kept with a cut-off value of [−log10(P value)< 3.0].

The additive genetic effect refers to the portion of phenotypic variation that can be attributed to the additive effects of multiple genetic loci. In the output, *R^2^* value explains how much of the phenotypic variation can be accounted for by the significant SNP marker. A higher *R^2^* value indicates a stronger relationship between the marker and the observed trait. While the positive or negative allele effect indicates whether the allele increases susceptibility or provides resistance to the disease, respectively. When the allele effect is positive, it suggests that possessing that allele increases the disease score, indicating susceptibility to the disease. Conversely, a negative allele effect signifies that the allele reduces the disease score, indicating resistance to the disease. The Manhattan plots of GWAS were generated using CMplot package in R 3.3.1.

### 2.7 QTL alignment and candidate genes

The marker sequences of previously reported QTL were retrieved from Grain Genes database (https://wheat.pw.usda.gov/blast/), and their positions were checked on the barley pseudomolecules Morex v. 2.0 2019 and Morex v3 pseudomolecules (2021) using Barleymap pipeline (Cantalapiedra et al., 2015). For candidate genes (CGs), the sequences of significant SNP markers were subject to BLAST search tool of IPK barley server (https://galaxy-web.ipk-gatersleben.de/) and based on a threshold of BIT score (> 200), sequence identity (90-100%), and an expect value (0 – 1^.40^). The candidate gene search considered the presence of proteins with functional domains implicated in plant disease resistance.

## 3 Results

### 3.1 Seedling resistance of AM2017 panel with three isolates of *R. commune*

Analyses of the phenotypic data showed a uniform disease establishment and diverse infection responses (IR) depending on the virulence spectrum of each isolate at the seedling stage under controlled conditions. The average IR was 0 and 5, respectively for the resistant (ICARDA 4 and Atlas 46) and susceptible (Tocada and Tissa) checks for all the three isolates.

**Figure 1:**
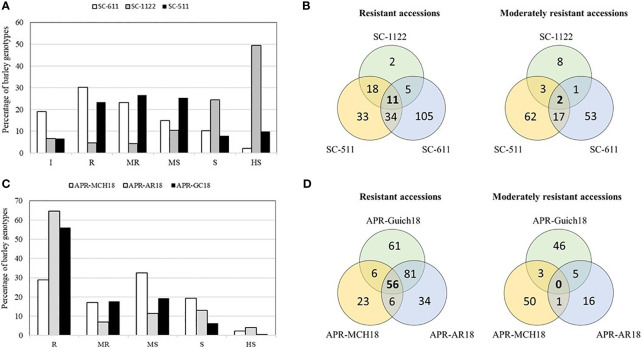
Frequency distribution of leaf scald resistance in 316 barley genotypes of AM2017 mapping panel at the seedling stage against *R. commune* isolates, SC-511, SC-611, and SC-1122 **(A)**. Venn diagrams showing barley genotypes with combined resistance at the seedling stage to the three *R. commune* isolates under controlled conditions **(B)**. Frequency distribution of leaf scald resistance in 316 barley genotypes of AM2017 mapping panel at the adult plant stage at Marchouch (MCH18), Rommani (AR18), and Guich (Guich18) **(C)**. Venn diagrams showing barley genotypes with combined resistance at the adult plant stage at three field locations **(D)**.

An average IR of 1.52 for *Rc* isolate SC-611, 3.6 for isolate SC-1122, and 1.98 for isolate SC-511 was observed in the AM2017 panel. Moreover, the frequency distribution of IR of AM2017 panel followed a normal distribution for the *Rc* isolates SC-511 and SC-611, whereas the distribution was negatively skewed towards susceptibility for the isolate SC-1122 (**Figure 1A**). Furthermore, the highest percentages of resistant genotypes were observed for the *Rc* isolate SC-S611 with 19.1% (60) immune and 30.3% (95) resistant genotypes, followed by the *Rc* isolate SC-511 with 6.7% (22) immune and 23.4% (74) resistant genotypes, whereas the *Rc* isolate SC-1122 showed the highest cumulative percentage of 73.8% (233) of susceptible and highly susceptible genotypes (**Figure 1A**). The *Rc* isolate SC-511 showed the highest percentage of moderately resistant (23.2%) genotypes followed by SC-S611 and SC-1122 isolate with 23.2% and 4.4%, respectively. Six-row genotypes had higher percentage of resistant barley genotypes with an average IR of 1.25 ± 0.9 for *Rc* isolate SC-511, followed by IR of 1.4 ± 1.1 for SC-S611, and IR of 2.9 ± 1.5 for SC-1122, whereas, the two-row barley genotypes were prone to susceptibility with an average IR of 1.62 ± 1.3 for SC-S611, followed by 2.6 ± 1.1 for SC-S511, and 4.1 ± 1.2 for SC-1122 isolates. About 11 barley genotypes, AM5, AM19, AM39, AM51, AM53, AM59, AM165, AM221, AM248, AM267, and AM280 were resistant to all the three *Rc* isolates used (**Figure 1B**). The heritabilities of scald resistance for all the three isolates at the seedling stage ranged from 0.40 to 0.58 (**Table 1**).

**Table 1:** The heritability estimates of scald resistance and the response of 316 genotypes of AM2017 panel at the seedling stage against *R. commune* isolates, SC-511, SC-1122, and SC-611 at the adult plant stage at Marchouch (MCH18), Rommani (AR18), and Guich (Guich18).

	*Seedling stage resistance*			*Adult stage resistance*		
	SC-511	SC-1122	SC-611	APR-MCH18	APR-AR18	APR-Guich18
Number of R genotypes	96	36	155	91	204	177
% of R gentotypes	30.1%	11.3%	49.4%	28.8%	64.5%	56%
Number of MR genotypes	84	14	73	54	22	56
% of MR gentotypes	26.6%	4.4%	23.2%	17.1%	7%	17.8%
Number of MS genotypes	80	33	47	103	36	61
% of MS gentotypes	25.4%	10.5%	15%	32.6%	11.4%	19.3%
Number of S genotypes	56	233	39	68	54	22
% of S gentotypes	17.8%	73.8%	12.4%	21.5%	17.1%	6.9%
Range of severity	0-5	0-5	0-5	1-9	1-9	0-9
Genotypes evaluated	316	316	316	316	316	316

The interaction between barley differentials against the three *Rc* isolates showed that the *Rc* isolate SC-1122 was avirulent on Atlas (*Rrs2*), Atlas 46 (*Rrs1, Rrs2*), Brier (*Rrs1*), however it was virulent on CI3515 (*Rh4, Rh10*), and La Masita (*Rh10, Rh4*). The *Rc* isolate SC-511 was virulent on Atlas (*Rrs2*), Brier (*Rrs1*), CI4364 (*rh11*), and CI3515 (*Rh4, Rh10*), but it was avirulent on Atlas 46 (*Rrs1, Rrs2*), Jet (*Rh6, rh7, rh6*), Osiris (*Rh4, rh6, Rh10, BRR6*), and Turk (*Rh5, Rrs1_Turk_, rh6*). However, the *Rc* isolate SC-611 was avirulent on all of the differentials tested except Skiff. A differential interaction was observed for the *Rc* isolate SC-511 on Atlas, Brier and CI4364. It can be concluded that the *Rc* isolate SC-511 may be NIP1 deficient as it displayed compatible interaction on Brier (*Rrs1*) as reported previously (Stefansson et al., 2014; Mohd-Assaad et al., 2019). Interestingly, it also showed compatible interaction on Atlas (*Rrs2*). Furthermore, four differentials showed incompatible interaction against the three *Rc* isolates tested; Atlas 46 (*Rrs1, Rrs2*), Jet (*Rh6, rh7, rh, rh6*), Osiris (*Rh4, rh6, Rh10, BRR6*), and Turk (*Rh5, Rrs1Turk (Rh3), rh6*). A differential interaction was observed for the *Rc* isolate SC-511 on Atlas, Brier and CI4364. Furthermore, four differentials showed incompatible interaction against the three *Rc* isolates tested; Atlas 46 (*Rrs1, Rrs2*), Jet (*Rh6, rh7, rh, rh6*), Osiris (*Rh4, rh6, Rh10, BRR6*), and Turk (*Rh5, Rrs1Turk (Rh3), rh6*).

#### 3.1.1 Adult plant stage phenotyping of AM2017 panel

At the adult plant stage, the disease severity on barley genotypes differed based on the environmental conditions in each location and the local pathogen population (**Figure 1C**). At Marchouch (MCH18) and Rommani (AR18) in 2018, the average disease severity of 1 on the resistant (ICARDA4 and Atlas 46) and 9 on the susceptible (Tocada and Tissa) checks were observed. While at Guich in 2018 (Guich18), the average disease severity of 0 and 7 was observed on the resistant and susceptible checks respectively.

The highest percentage of scald resistant genotypes was observed at AR18 with 64.5% (204) of genotypes compared to 56% (177) at Guich18 and 28.8% (91) of resistant genotypes at MCH18 (**Figure 1C**). Likewise, the highest percentage of scald susceptible genotypes were found at MCH18 with 21.5% (68) of genotypes, followed by 17.1% (54) genotypes at AR18, and 6.9% (22) susceptible genotypes at Guich18. About 56 genotypes were resistant across locations, whereas only seven genotypes displayed combined resistance at the seedling and adult plant stages (**Figure 1D**; **Table 2**). The heritabilities of scald resistance at the adult plant stage for three environments ranged from 0.89 to 0.99 (**Table 1**).

**Table 2:** Immune (I) and resistant (R) barley genotypes in AM2017 mapping panel to three *R. commune* isolates at the seedling stage and at the adult plant stage at three field locations in Morocco.

Line #	Pedigree	Row type	SRT- SC511	SRT- SC1122	SRT- SC611	APR-AR18	APR- Guich18	APR- MCH18
AM5	ICARDA SN326	6	I	I	I	R	I	R
AM39	M104/PFC 88210//DOÑA JOSEFA	6	I	R	I	R	R	R
AM51	HB511/JAEGER H00056005 09/3H0078	6	R	I	I	R	I	R
AM53	P.STO/3/LBIRAN/UNA80//LIGNEE640/4/B LLU/5/PETUNIA 1/6/CHAMICO/TOCTE//CONGONA	6	I	R	I	R	R	R
AM59	MSEL/FNC1	2	R	I	I	R	I	R
AM248	Isaria	6	I	I	R	R	I	R
AM280	15UCM 92	2	I	I	I	R	I	R

#### 3.1.2 Population structure and linkage disequilibrium

**Figure 2:**
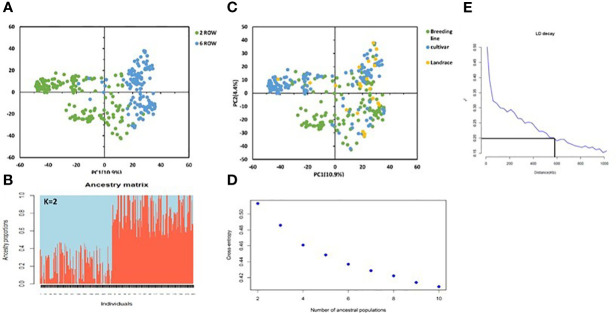
Principal component analysis (PCA) using 36,793 SNP markers displaying the spatial distribution of the 316 genotypes **(A, B)**. Genotypes are shown with colored round symbols based on their germplasm type **(A)** and row type **(B)**. Population structure using sNMF fonction at K=2 **(C)**. Values of the cross-entropy criterion for SNMF algorithm to evaluate several putative populations (K) ranging from K=2 to K=10 **(D)**. Linkage disequilibrium plot where intra-chromosomal values were plotted against the physical genetic distance **(E)**.

We performed principal component analysis (PCA) using a filtered set of 36,793 SNP markers to evaluate the genetic diversity of the 316 barley genotypes. The PCA of AM2017 categorized the panel into distinct clusters and showed a clear differentiation based on row type and germplasm type. About 10.6% of the genotypic variance was explained in the first axis of the PCA, partitioned the genotypes into two clearly defined groups according to their row type, being the six-rowed genotypes located towards the positive side of the axis and the two-rowed genotypes in the negative side (**Figure 2A**). The same separation was observed using sNMF, cross-validation and the cross-entropy criterion approaches, indicating that the AM2017 panel at K=2 is partitioned into two major clusters based of row types (**Figure 2B**). About 40.18% of the two-row genotypes were represented by one cluster and 59.81% of the six-row genotypes were represented by the second cluster. In addition, the PCA of the AM2017 showed a separation according to the germplasm type, but the barley landraces were not grouped into distinct group (**Figure 2C**). The cross-entropy criterion did not show a clear plateau and steadily decreased at higher K values. However, significant decreases in the criterion were observed from 2 to 10 (**Figure 2D**). Furthermore, the LD decay of AM2017 was estimated to be ~600 kb (~0.4 cM) (r^2 = ^0.2; **Figure 2E**).

### 3.2 GWAS of scald resistance at the seedling stage

**Figure 3:**
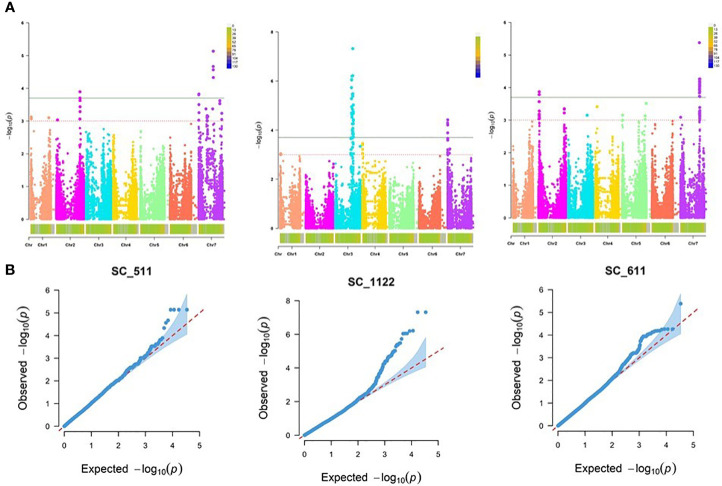
Genome-wide association mapping of barley scald resistance at the seedling stage. **(A)** The Manhattan plots display significant marker-trait associations on all chromosomes of barley at -log_10_ of p-values (LOD 3.0 and 3.7 for *R. commune* isolates SC-511, SC-1122, and SC-611. **(B)** Quantile-Quantile (Q-Q) plots of marker-trait associations at the seedling stage using MLM + PCA + K model.

Performing GWAS for the assessment of resistance to the three *R. commune* isolates, SC-511, SC-1122 and SC-611 at the seedling stage, the best fitting model was the MLM procedure using PCA, accounting for population structure (Q) and relatedness (K) (**Figures 3A, B**).

Our genome scan detected 102 significant MTAs corresponding to 15 QTL against the three *Rc* isolates at the seedling stage, with a range of marker *R^2^* from 3.55 – 9.84%, and additive effects ranging from -1.62 to 1.45 (**Table 3**). In addition, 14 (93%) out of 15, and 6 (50%) out of 12 APR QTL could be validated by more than 3 models. The genome scan for *Rc* isolate SC-511 detected 15 MTA corresponding to 5 QTL on chromosomes 2H and 7H, with marker *R^2^* and the additive effect ranging from 3.55 to 6.64%, and from -1.02 to 0.905, respectively. The highest phenotypic variation of 6.64% was explained by the SNP marker JHI-Hv50k-2016-483776 associated with *qSc.SRT4* on 7H (390.164 Mb; 70.54 cM), reducing the disease severity by -0.886 (17.2%), and by the SNP marker SCRI_RS_226361 associated with qSc.SRT3 on 7H (5.168 Mb; 1.91 cM), reducing the disease severity by -0.637 (21%) and explaining 4.69% of phenotypic variation.

**Table 3:** Summary of QTL associated with resistance against three isolates of *Rhynchosporium commune* in barley AM2017 panel at the seedling stage.

QTL	Peak SNP	^a^Chr	^b^QTL interval (Mb)	# of MTAs in QTL interval	*P-value*	Marker *R^2^* (%)	Allele frequency	^c^Allele Effect	TASSEL and GAPIT models
*SRT-SC-511									
*qSc.SRT1*	SCRI_RS_155612	2	27.65	1	9.27E-04	3.55	209	G (-0.504)	MLM(K+PCA),MLM(K+Q),MMLM
***qSc.SRT2***	JHI-Hv50k-2016-107864	2	579.22-583.15	2	1.28E-04	4.79	172	A (0.905)	MLM(K+PCA),MLM(K+Q),MMLM
***qSc.SRT3***	SCRI_RS_226361	7	4.83-10.86	2	1.58E-04	4.69	71	A (-0.637)	MLM(K+PCA),MLM(K+Q),MMLM,FarmCPU,SUPER
***qSc.SRT4***	JHI-Hv50k-2016-483776	7	390.06-393.2	7	7.29E-06	6.64	186	C (-0.886)	MLM(K+PCA),MLM(K+Q),MMLM
*qSc.SRT5*	JHI-Hv50k-2016-508670	7	614.71-615.55	3	5.82E-04	3.83	300	A (-1.02)	MLM(K+PCA),MLM(K+Q),MMLM,FarmCPU,BLINK,SUPER
*SRT-SC-1122									
***qSc.SRT6***	JHI-Hv50k-2016-180055	3	398.56-400.14	3	2.07E-04	4.47	36	C (-1.17)	MLM(K+PCA),MLM(K+Q)
***qSc.SRT7***	JHI-Hv50k-2016-180771	3	408.13-412.97	6	1.86E-06	7.55	27	C (-1.6)	MLM(K+PCA),MLM(K+Q),MMLM
***qSc.SRT8***	JHI-Hv50k-2016-181208	3	414-38-421.30	6	8.81E-07	7.99	29	A (-1.56)	MLM(K+PCA),MLM(K+Q),MMLM
***qSc.SRT9***	JHI-Hv50k-2016-182481	3	429.53-434.62	5	3.49E-06	7.08	290	C (1.45)	MLM(K+PCA),MLM(K+Q),MMLM
***qSc.SRT10***	JHI-Hv50k-2016-182733	3	438.46-441.69	12	4.78E-08	9.94	31	C (-1.62)	MLM(K+PCA),MLM(K+Q),MMLM,BLINK,FarmCPU
***qSc.SRT11***	JHI-Hv50k-2016-183825	3	445.97-454.93	17	1.78E-05	6.03	24	C (-1.5)	MLM(K+PCA),MLM(K+Q),MMLM,FarmCPU
***qSc.SRT12***	JHI-Hv50k-2016-434888	7	1.97-6.02	6	5.13E-05	5.37	271	A (1.08)	MLM(K+PCA),MLM(K+Q),MMLM,FarmCPU
*SRT-SC-611									
***qSc.SRT13***	JHI-Hv50k-2016-71369	2	17.76-19.21	2	1.35E-04	5.27	64	C (-0.775)	MLM(K+PCA),MLM(K+Q),MMLM,SUPER
*qSc.SRT14*	JHI-Hv50k-2016-443516	7	7.66	1	8.22E-04	3.64	286	C (-0.88)	MLM(K+PCA),MLM(K+Q),MMLM,SUPER
***qSc.SRT15***	JHI-Hv50k-2016-490465	7	508.91-521.12	29	4.17E-06	7.05	181	A (-0.798)	MLM(K+PCA),MLM(K+Q),MMLM,SUPER

Rhynchosporium commune isolate, SRT-SC-511, SRT-SC-1122, and SRT-SC-611.

a Chromosome.

b Positions on the barley pseudomolecules Morex v. 2.0 2019.

c Allele effect contributed by the respective marker on a 0–5 scale at the seedling stage. The negative allele effect decreases the diseases severity (resistance) and the positive allele effect increases the diseases severity (susceptibility).

The QTL indicated in bold text were significant with Bonferroni correction.

**Figure 4:**
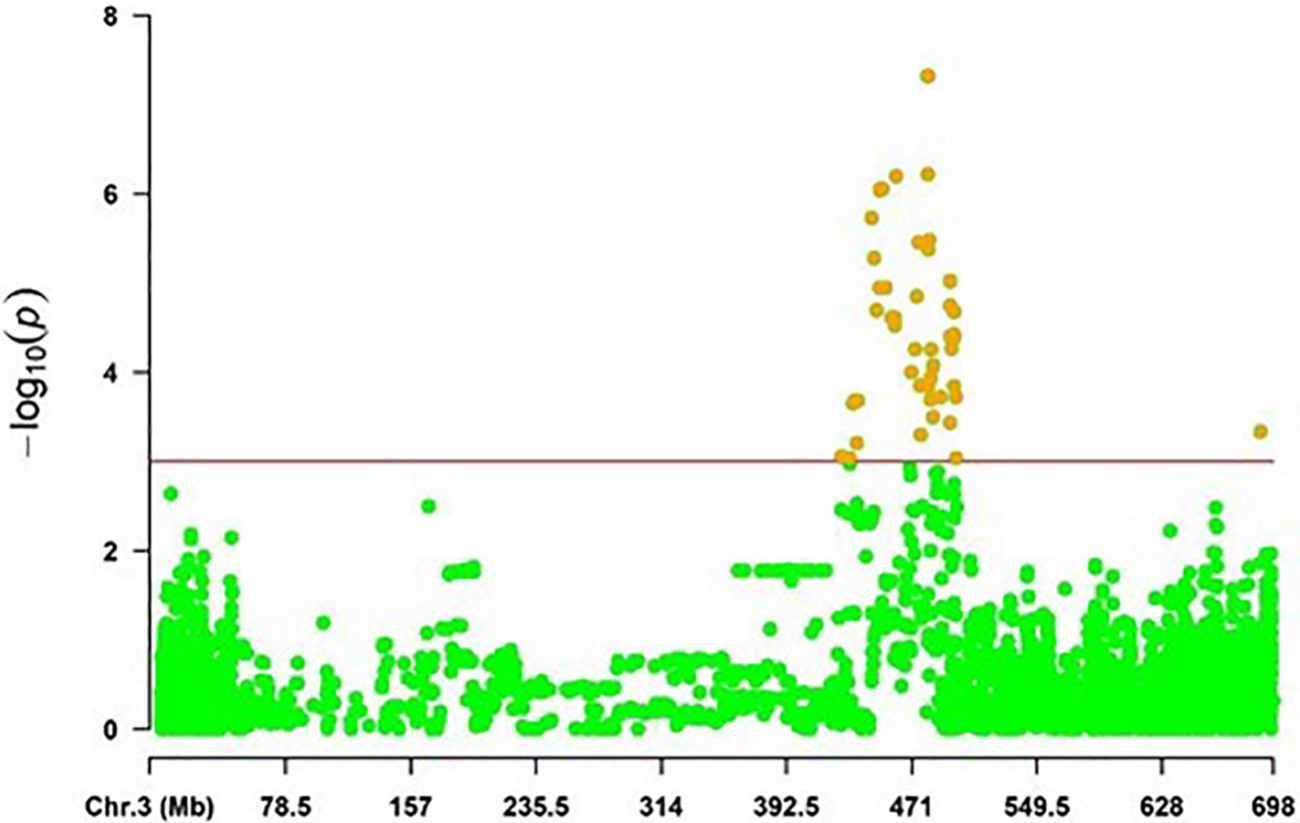
The distribution of significant SNP markers associated with scald resistance at the seedling stage on the chromosome 3H in a region from 400 to 500 Mb.

For *Rc* isolate SC-1122, 55 MTAs corresponding to 7 QTL, were detected on chromosomes 3H and 7H, with marker *R^2^* and the additive effect ranging from 4.47 to 9.94%, and from -1.62 to 1.45, respectively. The highest phenotypic variation of 9.94% was explained by the SNP marker JHI-Hv50k-2016-182733 associated with *qSC.SRT10* on 3H (438.945 Mb; 51.63 cM), reducing the disease severity by -1.62 (32.4%). In addition, the results demonstrated that several genomic regions were mapped at the vicinity of the previously mapped alleles loci on chromosome 3H (**Figure 4**).

Likewise, for *Rc* isolate SC-611, 32 MTAs represented by 3 QTL were detected on chromosomes 2H and 7H, with marker *R^2^* and the additive effect ranging from 3.64 to 7.05%, and from -0.88 to -0.775, respectively (**Table 3**). The highest phenotypic variation of 7.05% was explained by a SNP marker JHI-Hv50k-2016-490465 associated with *qSc.SRT15* on 7H (508.91 Mb; 77.41 cM). However, the highest reduction in disease severity of -0.88 (17.6%) was caused by the SNP marker JHI-Hv50k-2016-443516 associated with *qSc.SRT14* on 7H (7.666 Mb; 3.97 cM).

### 3.3 GWAS of scald resistance at the adult stage

**Figure 5:**
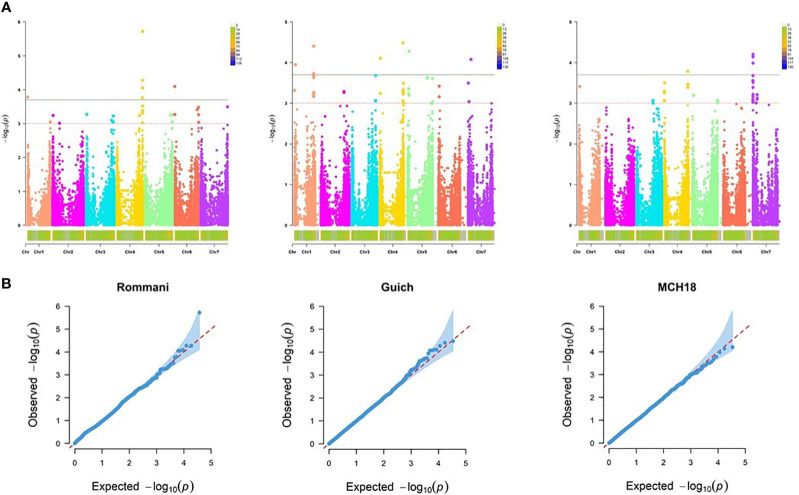
Genome-wide association mapping of barley scald resistance at the adult plant stage. **(A)** The Manhattan plots display significant marker-trait associations on all chromosomes of barley at -log_10_ of p-values (LOD 3.0 and 3.7 for three field locations: Marchouch (APR-MCH), Guich (APR-Guich18), and Rommani (APR-Rommani18). **(B)** Quantile-Quantile (Q- Q) plots of marker-trait associations at the adult plant stage using MLM + PCA + K model.

For the resistance at the adult plant stage, MLM procedure accounting for population structure (Q or PCA) and relatedness (K) was identified as the best fitting model (Q-Q plots for all tested models are shown in **Figure 5**). Our genome scan detected 25 significant MTA corresponding to 12 QTL scattered on all barley chromosomes except the chromosome 2H, with a marker *R^2^* ranging from 4.10 – 6.42%, and additive effects from -2.25 to -1.070 (**Table 4**). In addition, 19 MTAs, corresponding to 16 QTL with a LOD score of 3.0 – 3.6 have been presented in **Supplementary Table S1** as they had been reported in previous studies, thus validating our analysis.

**Table 4:** Summary of QTL associated with *Rhynchosporium commune* resistance in barley AM2017 panel at the adult plant stage.

QTL	Peak SNP	^a^Chr	^b^QTL interval (Mb)	# of MTAs in QTL interval	*P-value*	Marker *R^2^* (%)	Allele frequency	^c^Allele Effect	TASSEL and GAPIT models
*APR-MCH18									
***qSc.APR1***	JHI-Hv50k-2016-267965	4	605.577	1	1.64E-04	4.76	134	C (-7.8)	MLM(K+PCA),MLM(K+Q),MMLM,BLINK
***qSc.APR2***	JHI-Hv50k-2016-440870	7	1.96-4.86	4	1.03E-04	4.91	55	A (-9.03)	MLM(K+PCA),MLM(K+Q),BLINK,FarmCPU
*APR-Guich18									
***qSc.APR3***	JHI-Hv50k-2016-43566	1	484.55-485.81	4	3.94E-05	5.56	40	C (-1.8387)	MLM(K+PCA),MLM(K+Q),MMLM
***qSc.APR4***	JHI-Hv50k-2016-206686	3	578.71	1	2.09E-04	4.47	260	A (-1.4852)	MLM(K+PCA),MLM(K+Q),MMLM,FarmCPU,BLINK
***qSc.APR5***	JHI-Hv50k-2016-231680	4	19.77-19.78	2	7.86E-05	5.08	95	A (-1.30166)	MLM(K+PCA),MLM(K+Q),MMLM,BLINK
***qSc.APR6***	JHI-Hv50k-2016-265990	4	602.22	1	3.30E-05	5.63	81	A (-1.42184)	MLM(K+PCA),MLM(K+Q),MMLM,FarmCPU,BLINK
***qSc.APR7***	SCRI_RS_114729	5	37.99	1	5.31E-05	5.35	163.00	A (1.2627)	MLM(K+PCA),MLM(K+Q),MMLM
***qSc.APR8***	JHI-Hv50k-2016-469294	7	75.124	2	8.39E-05	5.06	279	C (-1.9035)	MLM(K+PCA),MLM(K+Q),MMLM
*APR-Rommani-18									
***qSc.APR9***	JHI-Hv50k-2016-4445	1	4.89-5.05	1	2.68E-05	5.77	296	C (-2.2502)	MLM(K+PCA),MLM(K+Q),MMLM
***qSc.APR10***	JHI-Hv50k-2016-254564	4	539.411-540.66	2	1.10E-04	4.87	34	G (1.6833)	MLM(K+PCA),MLM(K+Q),MMLM,BLINK
***qSc.APR11***	SCRI_RS_179398	4	606.16-608.35	5	9.69E-06	6.42	221	A (-1.2878)	MLM(K+PCA),MLM(K+Q),MMLM,BLINK,FarmCPU
***qSc.APR12***	JHI-Hv50k-2016-433362	6	571.58	1	1.07E-04	4.94	119	A (-1.35513)	MLM(K+PCA),MLM(K+Q),MMLM

*Environment and cropping season; APR-MCH (Marchouch), APR-Guich (Guich), APR-Rommani (Rommani).

^a^ Chromosome.

^b^ Positions on the barley pseudomolecules Morex v. 2.0 2019.

^c^ Allele effect contributed by the respective marker on a 0–9 scale at the adult plant stage. The negative allele effect decreases the diseases severity (resistance) and the positive allele effect increases the diseases severity (susceptibility).

The QTL indicated in bold text were significant with Bonferroni correction.

In case of Rommani site (AR18), 9 MTA represented by 4 QTL on the chromosomes 1H, 4H, and 6H were involved in scald disease resistance with a range of marker *R^2^* of 4.55 to 6.42, and additive effect of -2.25 to -1.107. The highest phenotypic variation of 6.42% was explained by a SNP marker SCRI_RS_179398 associated with *qSc.APR11* on 4H (606.21 Mb; 103.9 cM), reducing the disease severity by -1.28 (14.2%). However, the highest reduction in disease severity of -2.25 (25%) was caused by a SNP marker JHI-Hv50k-2016-4445 associated with *qSc.APR9* on 1H (5.04 Mb; 4.96 cM), explaining a phenotypic variation of 5.7%. For Guich (Guich18), 11 MTA represented by 6 QTL were detected on all barley chromosomes except 2H and 6H with a range of marker *R^2^* of 4.10 to 5.63, and additive effect of -1.903 to -1.262 (**Table 4**). The highest phenotypic variation of 5.63% was explained by the SNP marker JHI-Hv50k-2016-265990 associated with *qSc.APR6* on 4H (602.22 Mb; 99.43 cM), reducing the disease severity by -1.42 (15.7%). However, the highest reduction in disease severity of -1.9 (21.1%) was attributed to the SNP marker JHI-Hv50k-2016-469294 associated with *qSc.APR8* on 7H (75.12 Mb; 61.47 cM), explaining a phenotypic variation of 5.06%. For Marchouch (MCH18), 5 MTA represented by 2 QTL were detected on the chromosomes 4H and 7H with a range of marker *R^2^* 4.51 to 5.58 and additive effects from -9.03 to -7.22. The highest phenotypic variation of 5.58% was caused by the SNP marker JHI-Hv50k-2016-438638 associated with *qSc.APR2* on 7H (3.376 Mb; 1.63 cM). However, the highest reduction in disease severity of -9.03 was caused by the SNP marker JHI-Hv50k-2016-440870 associated with *qSc.APR2* on 7H (4.83 Mb; 52.01 cM). Interestingly, 4 SNP markers were associated with *Rc* resistance at both the seedling and the adult plant stages (**Table 5**).

**Table 5:** A list of common SNP markers and their encoded candidate genes for the seedling and adult plant stage resistance to *Rhynchosporium commune* in barley AM2017 panel.

QTL	Marker	^a^Chr	Environments (^c^Allele effect)	^b^Position Morex V2 (Mbs)	Candidate gene	References
***qSc.SRT7, qSc.APR13***	JHI-Hv50k-2016-180771	3	SC-112 (-1.6) MCH18 (-11.34)	408.1375	CTP synthase family protein	Looseley et al. (2015)
**qSc.SRT21, qSc.APR2**	JHI-Hv50k-2016-438980	7	SC-112 (0.691) MCH18 (7.7)	3.641	NBS-LRR disease resistance-like protein	Bjørnstad et al. (2004); Thauvin et al. (2022)
**qSc.SRT3, qSc.SRT12, qSc.APR2**	JHI-Hv50k-2016-440870	7	SC-511 (-0.6) SC-1122 (-0.911) MCH18 (-9.3)	4.833	LRR receptor-like serine/threonine-protein kinase EFR, Disease resistance protein (CC-NBS-LRR class) family	Bjørnstad et al. (2004); Thauvin et al. (2022)
**qSc.SRT3, qSc.SRT12**	SCRI_RS_226361	7	SC-511 (-0.637) SC1122 (-0.692)	4.8364	LRR receptor-like serine/threonine-protein kinase EFR, Disease resistance protein (CC-NBS-LRR class) family	Bjørnstad et al. (2004); Thauvin et al. (2022)

^a^ Chromosome.

^b^ Positions on the barley pseudomolecules Morex v. 2.0 2019.

^c^ Allele effect contributed by the respective marker on a 0–5 scale at the seedling stage. The negative allele effect decreases the diseases severity (resistance) and the positive allele effect increases the diseases severity (susceptibility).

The QTL indicated in bold text were significant with Bonferroni correction.

### 3.4 Candidate genes and QTL alignment

Based on the annotation of the sequences in the barley reference genome, the majority of the MTAs were located in genomic regions that are enriched with functional proteins associated with plant disease resistance and defense mechanisms. The BLAST searches revealed their homology with functional proteins/enzymes implicated in host plant disease resistance such as Serine/threonine-protein kinase Receptor-like protein kinase family protein, Disease resistance protein (CC-NBS-LRR class) family, receptor like kinases, Glutathion S-transferase, Nodulin MtN21/EamA-like transporter family protein, Subtilisin-like protease, ABC transporter ATP-binding protein, wall associated kinase, Glycosyltransferase family 92 protein, Hydroxycinnamoyl-CoA shikimate/quinate hydroxycinnamoyl transferase, ankyrin repeat family protein, Pentatricopeptide repeat-containing protein, Sugar transporter protein, and WD repeat domain phosphoinositide-interacting protein. Among 15 SRT QTL, BLAST searches using sequences of peak SNP markers revealed the homology of 4 SNP markers with RLKs (receptor like kinases) and 2 SNP markers with NLR (Nucleotide-binding leucine repeat). Likewise, in case of APR, SNP markers associated with 7 QTL encode RLK (**Tables 6**, **7**; **Supplementary Table S2**).

**Table 6:** QTL alignment and candidate genes identified for the seedling resistance against three isolates of *Rhynchosporium commune* in barley AM2017 panel.

QTL	Peak SNP	Chr	Position Morex V2 (Mbs)	Gene identifier	Homology	References
SRT-SC-511						
*qSc.SRT1*	SCRI_RS_155612	2	27.6563	HORVU.MOREX.r3.2HG0109310	Disease resistance protein (CC-NBS-LRR class) family	Hofmann et al. (2013); Looseley et al. (2018)
***qSc.SRT2***	JHI-Hv50k-2016-107864	2	582.6740	HORVU.MOREX.r3.2HG0184950	DNA-directed RNA polymerase subunit beta	
***qSc.SRT3***	SCRI_RS_226361	7	4.8364	HORVU7Hr1G002700	Serine/threonine-protein kinase	Bjørnstad et al. (2004); Thauvin et al. (2022)
***qSc.SRT4***	JHI-Hv50k-2016-483776	7	390.1641	HORVU.MOREX.r3.7HG0700570	Protein ENHANCED DISEASE RESISTANCE 2-like	
*qSc.SRT5*	JHI-Hv50k-2016-508670	7	614.9417	HORVU7Hr1G113370	LRR-repeat protein At5g22700-like	Wang et al. (2014); Thauvin et al. (2022)
SRT-SC-1122						
***qSc.SRT6***	JHI-Hv50k-2016-180055	3	400.1422	HORVU.MOREX.r3.3HG0275610	Pentatricopeptide repeat-containing protein	
***qSc.SRT7***	JHI-Hv50k-2016-180771	3	408.1374	HORVU.MOREX.r3.3HG0276500	CTP synthase family protein	Looseley et al. (2015)
***qSc.SRT8***	JHI-Hv50k-2016-181208	3	414.3801	HORVU.MOREX.r3.3HG0277290	Plant regulator RWP-RK family protein	Coulter et al. (2019)
***qSc.SRT9***	JHI-Hv50k-2016-182481	3	431.5177	HORVU.MOREX.r3.3HG0279830	Cysteine/Histidine-rich C1 domain family protein	Looseley et al. (2015)
***qSc.SRT10***	JHI-Hv50k-2016-182733	3	438.9457	HORVU.MOREX.r3.3HG0280440	Beclin-1-like protein	Williams et al. (2003)
***qSc.SRT11***	JHI-Hv50k-2016-183825	3	451.3086	HORVU.MOREX.r3.3HG0282120	Leucine-rich repeat receptor-like protein kinase	Grønnerød et al. (2002); Patil et al. (2003); Hofmann et al. (2013); Bjørnstad et al. (2004); Graner and Tekauz (1996); Looseley et al. (2020); Wagner et al. (2008); Dyck and Schaller (1961); Goodwin (1990); Genger et al. (2003)
***qSc.SRT12***	JHI-Hv50k-2016-434888	7	1.9762	HORVU.MOREX.r3.7HG0635050	LRR receptor-like serine/threonine-protein kinase EFR	Wallwork et al. (2014); Cheong et al. (2006); Bjørnstad et al. (2004); Thauvin et al. (2022)
SRT-SC-611						
***qSc.SRT13***	JHI-Hv50k-2016-71369	2	19.2131			Hofmann et al. (2013); von Korff et al. (2005), Daba et al. (2019)
*qSc.SRT14*	JHI-Hv50k-2016-443516	7	7.6668	HORVU.MOREX.r3.7HG0639110	Guanine nucleotide-binding protein alpha-2 subunit	Wagner et al. (2008); Looseley et al. (2018); Looseley et al. (2012)
***qSc.SRT15***	JHI-Hv50k-2016-490465	7	508.9174	HORVU.MOREX.r3.7HG0716940	L-ascorbate oxidase	Looseley et al. (2012)

**Table 7:** QTL alignment and candidate genes identified for the adult plant stage resistance against *Rhynchosporium commune* in barley AM2017 panel.

QTL	Peak SNP	Chr	Position Morex V2 (Mbs)	Gene identifier	Homology	References
APR-MCH18						
*qSc.APR1*	JHI-Hv50k-2016-267965	4	605.7778	HORVU4Hr1G083720	bZIP transcription factor 27	Jensen et al. (2002); Zantinge et al. (2019)
*qSc.APR2*	JHI-Hv50k-2016-440870	7	4.8325	HORVU.MOREX.r3.7HG0637270	Nuclease, putative	Hanemann et al. (2009); Goodwin (1990); Daba et al. (2019); Thauvin et al. (2022); Bjørnstad et al. (2004); Wallwork et al. (2014); Cheong et al. (2006)
	JHI-Hv50k-2016-439553	7	4.002	HORVU.MOREX.r3.7HG0636590	TSL-kinase interacting protein 1	
	JHI-Hv50k-2016-438638	7	3.376	HORVU7Hr1G001550	Very-long-chain 3-oxoacyl-CoA reductase 1	
	JHI-Hv50k-2016-438980	7	3.641	HORVU7Hr1G001750	NBS-LRR disease resistance-like protein	
APR-Guich18						
*qSc.APR3*	JHI-Hv50k-2016-43566	1	484.5590	HORVU.MOREX.r3.1HG0078000	Transmembrane protein 56-B	
	JHI-Hv50k-2016-43993	1	485.779	HORVU.MOREX.r3.1HG0078450	Ubiquitin carboxyl-terminal hydrolase 25	
	SCRI_RS_235724	1	485.809	HORVU.MOREX.r3.1HG0078480	FAR1-related sequence 5	
	JHI-Hv50k-2016-44088	1	485.810	HORVU.MOREX.r3.1HG0078480	FAR1-related sequence 5	
*qSc.APR4*	JHI-Hv50k-2016-206686	3	578.7114	HORVU.MOREX.r3.3HG0309560	Selenium-binding protein	Grønnerød et al. (2002), Loosely et al. (2015)
*qSc.APR5*	JHI-Hv50k-2016-231680	4	19.7783	HORVU.MOREX.r3.4HG0337450	Pentatricopeptide repeat-containing protein	
	JHI-Hv50k-2016-231677	4	19.78	HORVU.MOREX.r3.4HG0337450	Pentatricopeptide repeat-containing protein	
*qSc.APR6*	JHI-Hv50k-2016-265990	4	602.2248	HORVU.MOREX.r3.4HG0410190	Receptor-like protein kinase 4	Zantinge et al. (2019); Jensen et al. (2002)
*qSc.APR7*	SCRI_RS_114729	5	37.9994	HORVU.MOREX.r3.5HG0432280	Kinase interacting (KIP1-like) family protein	
*qSc.APR8*	JHI-Hv50k-2016-469294	7	75.1247	HORVU.MOREX.r3.7HG0663810	Aldose 1-epimerase, Fucosyltransferase	
	JHI-Hv50k-2016-469307	7	75.127	HORVU.MOREX.r3.7HG0663810	Aldose 1-epimerase, Fucosyltransferase	
APR-Rommani-18						
*qSc.APR9*	JHI-Hv50k-2016-4445	1	5.0419	HORVU.MOREX.r3.1HG0002370	Serine/threonine-protein kinase	Wang et al. (2014)
*qSc.APR10*	JHI-Hv50k-2016-254564	4	540.5062	HORVU.MOREX.r3.4HG0395710	Caleosin-related family protein	
	JHI-Hv50k-2016-254600	4	540.667			
*qSc.APR11*	SCRI_RS_179398	4	606.2172	HORVU.MOREX.r3.4HG0411620	Thyroid adenoma-associated protein homolog	Zantinge et al. (2019); Jensen et al. (2002); Looseley et al. (2012)
	JHI-Hv50k-2016-268095	4	606.168	HORVU.MOREX.r3.4HG0411600	Sugar transporter protein	
	JHI-Hv50k-2016-268154	4	606.21	HORVU.MOREX.r3.4HG0411620	Thyroid adenoma-associated protein homolog	
	JHI-Hv50k-2016-268158	4	606.212	HORVU.MOREX.r3.4HG0411620	Thyroid adenoma-associated protein homolog	
	JHI-Hv50k-2016-268616	4	608.352	HORVU.MOREX.r3.4HG0412330	Homeobox protein BEL1 homolog	
*qSc.APR12*	JHI-Hv50k-2016-433362	6	571.5888	HORVU.MOREX.r3.6HG0633580	Leucine-rich repeat domain superfamily	

Furthermore, 12 out of 15 SRT QTL, and 6 out of 12 APR QTL were validated by our study. However, 9 QTL on chromosomes 1, 2, 3, 4, 5, 6 and 7H reported in this study could be considered as novel (**Tables 3**, **4**).

## 4 Discussion

*Rhynchosporium commune* is one of the most destructive diseases of barley worldwide including in parts of North Africa and West Asia, requiring a continuous search for new sources of resistance and the development of associated molecular markers to introgress resistance loci to develop resistant varieties. In this study, we are reporting sources of resistance to *Rhyncosporium commue* from a collection of diverse barley genotypes at the seedling and at the adult plant stages. To our knowledge, this is the first GWAS study of scald resistance in spring barley from North Africa.

The successful inoculations at greenhouse and at the fields have allowed for efficient screening of AM2017 to scald at seedling and adult plant stages. A large number of sources of resistance were identified with differences in infection responses observed among the three *Rhynchosporium commune* isolates at the seedling stage and the at the three locations at the adult plant stage. Among the isolates, SC-1122 at the seedling stage, and the adult plant stage testing location APR MCH18 appeared to be the most virulent suggesting their use for further screening of germplasm. The high aggressiveness of SC-1122 could be due to the presence of functional NIP1 gene which affects virulence as reported by several authors (Stefansson et al., 2014; Mohd-Assaad et al., 2019).

The results confirmed the usefulness of AM2017 constructed by breeders in supplying sources of resistance to scald and to leaf rust (Amouzoune et al., 2022). In general, breeders prefer to use as parents, either elite lines or released varieties from different countries included in the constructed panels. However, core collections and other subsets of landraces conserved in genebanks and accessions of wild *Hordeum* are also bringing sources of resistance (Hiddar et al., 2021).

The monitoring of virulence of the pathogen and the screening of germplasm against the most virulent isolates and field populations could allow for a strategic deployment of effective resistance genes as an important component of integrated management of the disease. Major genes can be either used sequentially or pyramided. However, the screening of the same material at both seedling and adult plant stages could allow the identification of both minor and major genes of resistance which can be deployed for a more durable resistance protecting barley at all stages. However, compilation of these genes require the identification of QTLs associated with resistance.

Our results showed differences in infection response within the genotypes of AM2017 at the seedling stage to the three scald isolates. These differences could be attributed to diverse virulence spectrum of each *Rc* isolate. This notion was reinforced by conducting a race analysis of the three *Rc* isolates using barley differentials with known resistance genes. Based on the race analysis, it can be postulated that the *Rc* isolate SC-511 is NIP1 deficient as it showed compatible reactions both on Brier (*Rrs1*) and on Atlas (*Rrs2*).

Successful barley improvement depends on availability of genetic variation for breeders sought traits. Hence, with low genetic diversity in a given gene pool, crops become more vulnerable to biotic and abiotic stress (Sharma, 2017). The availability of the whole genome sequences and genotyping platforms have accelerated the identification, and the use of existing allelic diversity in barley germplasm. Compared with bi-parental mapping which is limited by allelic diversity among parents, GWAS can explore the existing allelic diversity and historical recombination events, resulting in reduced LD and superior mapping resolution. The LD of AM2017 panel was estimated to be 0.6 Mb (~0.4 cM), compared to earlier studies where LD of 3 to 5 cM was reported using different set of barley germplasm (Amezrou et al., 2018; Visioni et al., 2018; Visioni et al., 2020). In addition, AM2017 has an average SNP density of 37.09 SNPs/cM, which is much higher than earlier GWAS studies with 5 SNP/cM (Amezrou et al., 2018), 1 DArT marker per 1.5 cM (Comadran et al., 2009), and 1 SNP marker per 0.72 cM (Cockram et al., 2008; Pasam et al., 2012). Higher genetic diversity found in AM2017 is due to diverse origin of barley genotypes; 134 advance breeding lines from ICARDA’s international spring barley breeding program, 161 registered cultivars from Asia, Africa, Europe, America, and 21 landraces. In addition, very high heritability estimates were observed for scald resistance at the adult plant stage (0.89-0.99) compared with heritability estimates at the seedling stage (0.40 to 0.58). Similarly, Feriani et al. (2012) reported a higher heritability of 64% for all scald isolates. Likewise, Xi et al. (2000) also reported high heritability of scald resistance ranging from 0.57 to 0.91 under field conditions.

Barley QTL conferring resistance to *Rhynchosporium commune* at the seedling stage have been identified through linkage analysis of biparental mapping populations (Zhang et al., 2020). Though bi-parental mapping has resulted in the identification of eleven major scald disease resistance genes, *Rrs14* on chromosome 1H; *Rrs*1, *Rrs3*, and *Rrs4* on 3H; *Rrs6* and *Rrs9* on 4H; *Rrs13* on 6H; *Rrs2*, and *Rrs12* on 7H, most of the mapping studies converged on loci proximal of *Rrs1* (3H) and *Rrs2* (7H) (Zhang et al., 2020). Interestingly, the necrosis inducing protein (NIP1) is the product of avirulence gene *AvrRrs1* and conditions disease resistance in barley genotypes carrying *Rrs1*(Rohe et al., 1995; Steiner-Lange et al., 2003; Stukenbrock and McDonald, 2009). Based on the position of flanking markers on Morex genome V2, *Rrs1* has been mapped on 445-455 Mbs on the chromosome 3H, and we also detected 15 SNPs in the same genomic region for *Rc* isolate SC-1122 as reported by numerous studies (Dyck and Schaller, 1961; Goodwin, 1990; Graner and Tekauz, 1996; Grønnerød et al., 2002; Genger et al., 2003; Patil et al., 2003; Wagner et al., 2008; Hofmann et al., 2013; Looseley et al., 2020). Based on these findings, it can be postulated that only *Rc* isolate SC-1122 seems to have NIP1, whereas *Rc* isolates SC-611 and SC-511 did not harbor NIP1 as no QTL was detected on 3H. Likewise, *Rrs2* has been mapped on 5.2 Mb on 7H, and our genome scan has detected three QTL in the same genomic region; *qSc.SRT3* (4.83 Mb) for *Rc* isolate SC-511, *qSc.SRT12* (1.97-6.02 Mb) for SC-1122, and *qSc.SRT14* (7.66 Mb) for *Rc* SC-611. The same genomic region has been reported previously on 5.3 Mb by Daba et al. (2019) on 6.42 Mb, and in many other studies (Grønnerød et al., 2002; Bjørnstad et al., 2004; Cheong et al., 2006; Yan et al., 2009; Wallwork et al., 2014). These results demonstrated that many new genomic regions were mapped at the vicinity of the previously mapped loci on chromosome 3H and 7H.

On chromosome 1H, the major resistance gene *Rrs14* introgressed from *Hordeum spontaneum* was mapped on 2.8 Mb (Garvin et al., 2000). We detected one APR QTL *qSc.APR9* (4.89-5.0 Mb) on the short arm of 1H which is in close proximity of *Rrs14* locus. Another APR QTL *qSc.APR3* on chromosome 1H at 484.55-485.81 Mb seems to be novel as it is located ~482 Mb away from *Rrs14* locus. Interestingly, the peak SNP JHI-Hv50k-2016-4445 (5.04 Mb) associated with the APR QTL *qSc.APR9* reduced the disease severity by 2.25 units (25%) and validated a QTL reported on 4.28 Mb by Wang et al. (2014).

On chromosome 2H, for *Rc* isolate SC-511 two SRT QTL *qSc.SRT1* (27.65 Mb), *qScSRT2* (579.22-583.15 Mb), and for *Rc* SC-611 one SRT QTL *qSc.SRT13* (17.76-19.21 Mb) were detected. Our study has validated a QTL reported by Daba et al. (2019) on 17.12 Mb and the same QTL was reported by Hofmann et al. (2013) and (von Korff et al., 2005). Likewise, one APR QTL *qSc.APR21* has been reported by Looseley et al. (2012, 2015) (**Supplementary Table S2**).

On chromosome 3H, 49 SRT MTAs (48.03%) were grouped into 6 SRT QTL, spanning a physical distance from 394 to 454.93 Mb. Of the 6 SRT QTL from this study, five SRT QTL; *qSc.SRT7, qSc.SRT8, qScSRT9, qScSRT10*, and *qScSRT11* have been reported previously in different studies (Dyck and Schaller, 1961; Goodwin, 1990; Graner and Tekauz, 1996; Grønnerød et al., 2002; Genger et al., 2003; Patil et al., 2003; Cheong et al., 2006; Wagner et al., 2008; Hofmann et al., 2013; Looseley et al., 2015; Looseley et al., 2018; Coulter et al., 2019; Looseley et al., 2020). Whereas the SRT QTL *qSc.SRT6* (398.56-400.14 Mb) was novel. Furthermore, 4 APR MTAs were classified into 4 QTL and none of them overlapped with the mapped interval of *Rrs1*, which may indicate that the other genomic regions on 3H are important in conditioning adult plant stage resistance to scald. Alternatively, the field populations of *Rc* are either *AvrRrs1* deficient or have a mutated version of *AvrRrs1*. This can further be supported from our seedling screening assay where one of three *Rc* isolates SC-1122 detected QTL in *Rrs1* mapped locus. Three APR QTL; *qSc.APR13* (408-414 Mb), *qSc.APR4* (578.7-578.8 Mb), and *qSc.APR23* (597.95 Mb) have been reported previously (Grønnerød et al., 2002; Cheong et al., 2006; Looseley et al., 2015).

On chromosome 4H, the major resistance gene *Rrs16* introgressed from *H. bulbosum* was mapped on 1.7 Mb (Pickering et al., 2006). Likewise, we detected two MTAs linked to *qSc.APR14* spanning a genomic region of 0.39-2.37 Mb with the peak SNP marker JHI-Hv50k-2016-225852 on 1.36 Mb, reducing the disease severity by 7.88 AUDPC units (**Supplementary Table S2**). *Rc* QTLs have been reported in the same genomic region (Pickering et al., 2006; Wallwork et al., 2014; Wang et al., 2014). Similarly, *qSC.APR1* (605.57-606.16 Mb), *qSc.APR6* (602.22 Mb), and *qSc.APR11* (606.16-608.35 Mb) have been reported by several authors (Jensen et al., 2002; Looseley et al., 2012; Zantinge et al., 2019).

Five APR QTL were detected on 5H conditioning resistance to scald at two field locations. The two QTL *qSc.APR17* (Guich18) and *qSc.APR24* (AR18) were located between 550.77 to 553.49 Mb may represent the same QTL, and Looseley et al. (2012) also reported the same genomic region to be associated with scald resistance (**Supplementary Table S2**). Likewise, Daba et al. (2019) mapped a QTL on 587.44 Mb which coincides with two APR QTL; *qSc.APR18* (586.95 Mb) and *qSc.APR25* (582-589 Mb) reducing the disease severity by 0.9 (10%) to 1.13 (14%) units. Of the 5 APR QTL, only 1 QTL (*qSc.APR7*; 37.99 Mb) was novel. Interestingly, the peak SNP JHI-Hv50k-2016-343816 (553.49 Mb) associated with *qSc.APR24* reduced the disease severity by 1.78 units (20%) and can be used as a functional marker in Marker Assisted Selection (MAS).

In previous studies, two major scald resistance genes, *Rrs13* (11.8 Mb) and *Rrs18* (25.6 Mb) were mapped on the chromosome 6H (Abbott et al., 1995; Read and Oram, 1995; Genger et al., 2003; Coulter et al., 2019). Among the three APR QTL identified on 6H, *qSc.APR19* (23.712-23.746 Mb) was detected in close proximity of *Rrs13* and Shtaya et al. (2006) also mapped a QTL on 21.3 Mb. Further, another APR QTL *qSc.APR26* (4.34 Mb) was detected by Jensen et al. (2002) from the cultivar Alexis, and by Cheong et al. (2006) from the cultivar Keel in two different studies. No SRT QTL was detected on 6H for the three *Rc* isolates tested.

Of the 102 MTAs at SRT, 48 (47%) were mapped on the chromosome 7H and constituted 6 QTL of which 5 QTL were validated by our study and 1 QTL was novel. The SRT QTL *qSc.SRT5* (614.71-615.55 Mb) may represent the same QTL reported by Wang et al. (2014) and Thauvin et al. (2022) on 7H.

The markers/QTL stable across environments would be ideal candidates for MAS (marker assisted selection) and genomic selection (GS). Two common SRT QTL were detected. We detected two SNPs JHI-Hv50k-2016-440870 (4.832 Mb) and SCRI_RS_226361 (4.863 Mb) associated with *qSc.SRT3* and *qSc.SRT12* against *Rc* SC-511 and SC-1122 on 7H, respectively and may represent the same QTL. About 9 significant SNPs were encompassing a genomic region of 1.97-10.86 Mb on 7H, conditioning resistance to all the three *Rc* isolates. These may represent different alleles of *Rrs2* or different resistance genes. In addition, seven common APR QTL were detected across environments. Seven SNPs encompassed a genomic region of 602.22-608.35 Mb on 4H representing *qSc.APR1* (APR-MCH18), *qSc.APR16* (Guich18), and *qSc.APR11* (AR18) which may represent the same QTL. Likewise, the close proximity of *qSc.APR17* (550.77 Mb; Guich18) with *qSc.APR24* (553.49 Mb; AR18), and *qSc.APR18* (586.95 Mb; Guich18) with *qSc.APR25* (582.53-589.44; AR18) on 5H most probably represent the same QTL. A list of 4 common SNPs were shared between SRT and APR. Similarly, *qSc.APR20* (10.12 Mb; Guich18) and *qSc.APR27* (10.74 Mb; AR18) represent the same QTL. These SNPs can be used for MAS and GS platforms to accelerate gene pyramiding efforts.

The understanding of host-pathogen interaction at genetic level is quite important for identifying and deploying scald resistance. Plants have a multilayered inducible defense system. Their first layer of defense is based on extracellular pattern recognition receptors (PRRs) which recognize conserved microbe/pathogen/damage-associated-molecular patterns (MAMPs/PAMPs/DAMPs). Mostly PRRs are membrane anchored receptors with an ecto-domain for ligand binding for example leucine-rich-repeat (LRR) domain or a chitin binding (LysM) domain (Miya et al., 2007) with or without an endo- kinase domain like in receptor like kinases (RLKs) or receptor-like proteins (RLPs) which require a co-receptor for cellular signaling. The activation of PRRs induces transcriptome reprogramming and biosynthesis of antimicrobial compounds to limit pathogen’s ingress. However, successful pathogens use avirulence gene products (effectors) to subvert host defense responses. The second layer of defense is initiated upon recognition of pathogen secreted effectors by nucleotide-binding leucine-rich repeat (NLR) receptors either directly (gene-for-gene model) or indirectly by guarding and sensing conformational changes in the host virulence targets of the effectors which results in a localized cell death, a phenomenon referred here as effector triggered immunity (ETI) (Jones and Dangl, 2006). *R. commune* is an hemibiotrophic pathogen of barley and a gene-for-gene interaction has been proposed with its host. Three necrosis inducing proteins (NIP1, NIP2, and NIP3) have been described from *Rc*, where NIP1 is expressed in spores, but NIP2 and NIP3 are expressed upon host infection (Kirsten et al., 2012). NIP1 serves the function of an effector by inducing leaf necrosis, and as an elicitor to induce *Rrs1* dependent defense response (Wevelsiep et al., 1993; Rohe et al., 1995; Steiner-Lange et al., 2003a). That is why NIP1 has been under positive selection as indicated by the presence of two paralogous and copy number variations. From a global collection of 614 *Rc* isolates, Schürch et al. (2004) reported absence of NIP1 deletion in 45% of the isolates, whereas, NIP2 and NIP3 were present in all isolates. Different *Rc* isolates either lacking NIP1 or mutated NIP1 can overcome cultivars carrying *Rrs1* (Stukenbrock and McDonald, 2009; Mohd-Assaad et al., 2019). Therefore, the identification and deployment of both qualitative and quantitative resistance genes offers durable control of this rapidly evolving pathogen. None of the major resistance genes against scald have been cloned and functionally characterized, hence the molecular mechanism of disease resistance conditioned by *Rrs1* and *Rrs2* remains elusive. *Rrs1* does not encode a receptor for NIP1 but triggers resistance response upon recognition of NIP1 by an unknown co-receptor. With the availability of barley genome and predicted protein repertoire, functional homology-based BLAST searches using marker sequences associated with mapped QTL will help in candidate gene identification. Interestingly, the QTL *qSc.SRT11* was co-located within *Rrs1* mapped region on the chromosome 3H which was reported in different studies (Dyck and Schaller, 1961; Goodwin, 1990; Graner and Tekauz, 1996; Bjørnstad et al., 2002; Grønnerød et al., 2002; Patil et al., 2003; Wagner et al., 2008; Hofmann et al., 2013; Looseley et al., 2020).

RLKs contain an ectodomain, a transmembrane spanning region, and an intracellular cytoplasmic kinase domain to relay the pathogen recognition signal (Tör et al., 2004). The SNP marker JHI-Hv50k-2016-124224 associated with *qSc.SRT15* on chromosome 2H (632.26 Mb) encodes a serine/threonine-protein kinase (HORVU.MOREX.r3.2HG0199700), reducing the disease severity by 1.35 units (27%). Likewise, the SNP marker JHI-Hv50k-2016-4445 associated with *qSc.APR9* on chromosome 1H (5.041 Mb) also encodes a serine/threonine-protein kinase (HORVU.MOREX.r3.1HG0002370), reducing the disease severity by 2.25 units (25%). The Serine/threonine protein kinases phosphorylate the OH group of two amino acids, serine or threonine of the target protein, and this protein phosphorylation process plays an important role in the disease resistance pathway. In wheat the *Pm21* locus conditions durable resistance to wheat powdery mildew (*B.graminis* f.sp. *tritici (Bgt)*) (He et al., 2022). A putative serine/threonine-protein kinase (*Stpk-V*) was cloned from the *Pm21* locus and its transient expression in a susceptible wheat variety Yangmati 158 rendered broad spectrum durable resistance to powdery mildew. Whereas virus induced gene silencing of *Stpk-V* in resistant wheat cultivar rendered it highly susceptible to *Bgt* (Chen et al., 1995; Cao et al., 2011). In barley, the leaf rust resistance gene *Rph22* encodes a lectin receptor like kinase and some of the RLKs may possibly be involved in basal defense if an analogy is to be drawn with other extracellular PTT receptors (Wang et al., 2019). Likewise, in rice, an RLK (*Xa21*) confers resistance to bacterial blast caused by *Xanthomonas oryzae* pv. *Oryzae* (Song et al., 1995; Tör et al., 2004). We found 6 APR QTL (*qSc.APR6, qSc.APR7, qSc.APR18, qSc.APR19, qSc.APR21,and qSc.APR24*), and 5 SRT (*qSc.SRT2, qSc.SRT3, qSc.SRT11, qSc.SRT12, qSc.SRT15*) where several SNP markers encode RLK as candidate genes. The co-location of candidate genes within families with known role in disease resistance such as RLK, NLR and wall associated proteins can help identify new allelic variants of existing genes or completely new resistance genes.

Sugar transporters mediate sugar transport and are one of important players in plant-pathogen interaction (Breia et al., 2021). A SNP marker JHI-Hv50k-2016-268095 is associated with a APR QTL *qSc.APR11* on 4H at 606.1682 Mb and encodes a sugar transporter (HORVU.MOREX.r3.4HG0411610). This SNP marker reduced the disease severity by ~20% in MCH18. Recently cloned what leaf rust resistance gene *Lr67* encodes a sugar transporter 13 (SAT13), which plays a crucial role in conditioning partial resistance to all wheat rusts and powdery mildew (Milne et al., 2019). Its susceptible variant (*Lr67sus*) is a functional sugar transporter and its expression is upregulated upon pathogen invasion, whereas its dominant variant (*LR67res*) is unable to transport sugar which is required by wheat rusts and powdery mildew. Likewise, *Xanthomonas oryzae* pv. *oryzae* secreted TAL (Transcription-activator like) effector upregulate the expression of a sugar transporter *Os SWEETY11* in rice (Chen et al., 2010). Similar findings have been reported for Arabidopsis-*Botrytis cinerea*, grape vine interaction with powdery mildew (*Erysiphe nectar*) and downy mildew (*Plamopara viticola*) (Hayes et al., 2010; Lemonnier et al., 2014). Sugar transporters are quite conserved in plant kingdom and a similar role in disease resistance can be envisioned for these transporters in barley-*Rhynchosporium commune* interactions.

The nodulin-like proteins belong to a group of aquaporins and they have been implicated in the transport of various solutes in plants in addition to their interaction with various plant pathogens. In barley, the sequences of two SNPs on 7H, JHI-Hv50k-2016-483974 (*qSc.SRT4*, 393.195 Mb) encode putative Early Nodulin-like protein. Interestingly, Gyawali et al. (2018) also reported a QTL (*Rcs-qtl-7H-32.81*) for spot blotch resistance in barley on 7H where the SNP marker 11_20162 encoded an Early Nodulin-93 like protein.

The ABC transporter proteins condition disease resistance by secreting the anti-fungal products into the apoplast like PEN3 in Arabidopsis against *B. cinerea* (He et al., 2019), and *Lr34* against wheat leaf rust (Krattinger et al., 2009). We found that ten adjacent SNP markers (453.535-453.764 Mb), in the interval of *qSc.SRT11* on 3H, were flanked by ABC transporter B protein (HORVU.MOREX.r3.3HG0282510). In wheat, *Lr34* provided resistance against wheat stem rust, stipe rust, and powdery mildew (Rinaldo et al., 2017) in addition to powdery mildew and rust resistance in barley (Boni et al., 2018), and against rice blast (Krattinger et al., 2016). We speculate an important role of ABC transporter proteins in resistance against *R. commune*.

Our study detected several SNP markers encoding functional proteins which have been involved in plant defense against diverse fungal pathogens. The newly detected QTLs can be applied in barley breeding program and the information could be employed by breeders for marker assisted selection, fine mapping, and gene cloning for improving resistance to scald disease.

## 5 Conclusion

The identification of diverse sources of resistance and molecular tagging of new genomic regions associated with resistance to *R. commune* is crucial for future marker-assisted selection and genomic selection for continuous improvement of barley germplasm. Our GWAS was efficient in genetic dissection of scald resistance. Several SNPs were located within already known QTL associated with scald disease in addition to the identification of novel seedling stage resistance QTL on the chromosomes 2H, 3H, and 7H, and the adult stage resistance QTL on the chromosomes 1H, 4H, 5H, 6H, and 7H. Durable scald resistance requires further understanding of *R. commune* pathogen population dynamics and efficient introgression of major and minor resistance genes using molecular markers.

## Data availability statement

The original contributions presented in the study are publicly available. This data can be found here: https://bms.icarda.org:59395/public/?db=Barley_AM17&pj=AM17.

## Author contributions

HH, AA, BB, formulated the research problem and designed the approaches. HH, MJ, SR, BB, raised experiments in fields and recorded phenotyping data. HH, SR, SG, performed statistical analysis of data, AA and SR planned and supervised research activity. HH, SR, wrote the initial draft of the paper All authors contributed to the article and approved the submitted version.
