# Supplementary material for: Identification of sources of resistance to scald (Rhynchosporium commune) and of related genomic regions using genome-wide association in a mapping panel of spring barley
Source: Front Plant Sci. 2023 Nov 28;14:1133404. doi: 10.3389/fpls.2023.1133404 (PMC10715060; doi:10.3389/fpls.2023.1133404)
Supplement: Supplementary file 1 [file Table_1.docx]

| **QTL** | **Peak SNP** | **^a^Chr** | **^b^QTL interval (Mb)** | **# of MTAs in QTL interval** | ***P-value*** | **Marker *R^2^* (%)** | **Allele frequency** | **^c^Allele Effect** | **TASSEL and GAPIT models** |
| --- | --- | --- | --- | --- | --- | --- | --- | --- | --- |
| ***APR-MCH** | | | | | | | | | |
| *qSc.APR13* | JHI-Hv50k-2016-180771 | 3 | 408.13 | 1 | 8.56E-04 | 3.73 | 27 | C (-11.34) | MLM(K+PCA),MLM(K+Q),MMLM,FarmCPU |
| *qSc.APR14* | JHI-Hv50k-2016-225852 | 4 | 0.39-2.37 | 6 | 5.55E-04 | 3.93 | 70 | C (-7.88) | MLM(K+PCA),MLM(K+Q),MMLM,BLINK |
| *qSc.APR15* | JHI-Hv50k-2016-229327 | 4 | 10.12 | 1 | 3.15E-04 | 4.21 | 228 | C (-7.91) | MLM(K+PCA),MLM(K+Q) |
| *qSc.APR16* | JHI-Hv50k-2016-470775 | 7 | 82.61 | 1 | 9.82E-04 | 3.52 | 253 | C (-8.33) | MLM(K+PCA),MLM(K+Q),BLINK,FarmCPU |
| ***APR-Guich18** | | | | | | | | | |
| *qSc.APR17* | BOPA1_1306-408 | 5 | 550.77 | 2 | 1.01E-03 | 3.50 | 28 | C (-1.6525) | MLM(K+PCA),MLM(K+Q),MMLM,BLINK |
| *qSc.APR18* | SCRI_RS_195241 | 5 | 586.95 | 1 | 9.98E-04 | 3.53 | 151 | C (-0.9001) | MLM(K+PCA),MLM(K+Q) |
| *qSc.APR19* | JHI-Hv50k-2016-379632 | 6 | 23.712-23.746 | 2 | 6.95E-04 | 3.73 | 129 | A (-1.1239) | MLM(K+PCA),MLM(K+Q),MMLM,BLINK |
| *qSc.APR20* | JHI-Hv50k-2016-446390 | 7 | 10.12 | 1 | 3.20E-04 | 4.20 | 67 | C (-1.1865) | MLM(K+PCA),MLM(K+Q),MMLM |
| ***APR-Rommani-18** | | | | | | | | | |
| *qSc.APR21* | JHI-Hv50k-2016-137285 | 2 | 656.46 | 1 | 3.32E-04 | 4.18 | 269 | A (-1.5897) | MLM(K+PCA),MLM(K+Q),MMLM |
| *qSc.APR22* | JHI-Hv50k-2016-163896 | 3 | 35.57 | 1 | 6.30E-04 | 3.79 | 265 | A (-1.3612) | MLM(K+PCA),MLM(K+Q),MMLM,BLINK |
| *qSc.APR23* | JHI-Hv50k-2016-213966 | 3 | 597.95 | 1 | 3.39E-04 | 4.17 | 76 | A (-1.19592) | MLM(K+PCA),MLM(K+Q),MMLM,FarmCPU |
| *qSc.APR24* | JHI-Hv50k-2016-343816 | 5 | 553.49 | 1 | 9.98E-04 | 3.51 | 21 | C (-1.78137) | MLM(K+PCA),MLM(K+Q),MMLM,BLINK,FarmCPU |
| *qSc.APR25* | JHI-Hv50k-2016-358258 | 5 | 582.53-589.44 | 4 | 4.06E-04 | 4.06 | 81 | G (-1.1395) | MLM(K+PCA),MLM(K+Q),MMLM,BLINK |
| *qSc.APR26* | JHI-Hv50k-2016-367980 | 6 | 4.34 | 1 | 6.36E-04 | 3.81 | 226 | G (-1.1983) | MLM(K+PCA),MLM(K+Q),MMLM,BLINK,FarmCPU |
| *qSc.APR27* | JHI-Hv50k-2016-446947 | 7 | 10.74 | 1 | 6.93E-04 | 3.73 | 252 | A (-1.11557) | MLM(K+PCA),MLM(K+Q),MMLM |
| *qSc.APR28* | JHI-Hv50k-2016-516264 | 7 | 627.55 | 1 | 6.12E-04 | 3.81 | 282 | C (-1.6092) | MLM(K+PCA),MLM(K+Q),MMLM,BLINK,FarmCPU |

**Supplementary Table S1.** Summary of QTL associated with *Rhynchosporium commune* resistance in barley AM2017 panel at the adult plant stage with a LOD score of 3.0 – 3.60.

*Environment and cropping season; APR-MCH (Marchouch), APR-Guich (Guich), APR-Rommani (Rommani)

**^a^** Chromosome

**^b^** Positions on the barley pseudomolecules Morex v. 2.0 2019

**^c^** Allele effect contributed by the respective marker on a 0–9 scale at the adult plant stage. The negative allele effect decreases the diseases severity (resistance) and the positive allele effect increases the diseases severity (susceptibility).

**Supplementary Table S2.** QTL alignment and candidate genes identified for the adult plant stage resistance against *Rhynchosporium commune* in barley AM2017 panel.

| **QTL** | **Peak SNP** | **Chr** | **Position Morex V2 (Mbs)** | **Gene identifier** | **Homology** | **References** |
| --- | --- | --- | --- | --- | --- | --- |
| **APR-MCH** | | | | | | |
| *qSc.APR13* | JHI-Hv50k-2016-180771 | 3 | 408.1374 | HORVU.MOREX.r3.3HG0276500 | CTP synthase family protein | Looseley et al. (2014) |
| *qSc.APR14* | JHI-Hv50k-2016-225852 | 4 | 1.3675 | HORVU.MOREX.r3.4HG0331660 | RING/U-box superfamily protein | Pickering et al. (2006), Wang et al. (2014), Wallwork et al. (2014) |
| *qSc.APR15* | JHI-Hv50k-2016-229327 | 4 | 10.1219 | HORVU4Hr1G083720 |  | Loosely et al. (2018) |
| *qSc.APR16* | JHI-Hv50k-2016-470775 | 7 | 82.6164 | HORVU.MOREX.r3.7HG0665310 | Serine palmitoyltransferase long chain base subunit | Daba et al. (2019) |
| **APR-Guich18** | | | | | | |
| *qSc.APR17* | BOPA1_1306-408 | 5 | 550.7732 | HORVU5Hr1G103330 | 40S ribosomal protein S12 | Looseley et al. (2012) |
| *qSc.APR18* | SCRI_RS_195241 | 5 | 586.9538 | HORVU.MOREX.r3.5HG0532220 | Protein kinase | Daba et al. (2019) |
| *qSc.APR19* | JHI-Hv50k-2016-379632 | 6 | 23.7464 | HORVU.MOREX.r3.6HG0549530 | Leucine-rich repeat receptor-like protein kinase family protein | Shtaya et al. (2006) |
| *qSc.APR20* | JHI-Hv50k-2016-446390 | 7 | 10.1295 | HORVU.MOREX.r3.7HG0640540 | Ozone-responsive stress related protein | Schweizer et al. (1995) |
| **APR-Rommani-18** | | | | | | |
| *qSc.APR21* | JHI-Hv50k-2016-137285 | 2 | 656.4652 | HORVU.MOREX.r3.2HG0209820 | Leucine-rich repeat receptor-like protein kinase family protein | Looseley et al. (2012), Looseley et al. (2014) |
| *qSc.APR22* | JHI-Hv50k-2016-163896 | 3 | 35.5713 | HORVU.MOREX.r3.3HG0234270 | Autophagy-related protein 18 | Li and Zhou (2011) |
| *qSc.APR23* | JHI-Hv50k-2016-213966 | 3 | 597.9550 | HORVU.MOREX.r3.3HG0316640 | Pentatricopeptide repeat (PPR) superfamily protein | Cheong et al. (2006) |
| *qSc.APR24* | JHI-Hv50k-2016-343816 | 5 | 553.4967 | HORVU.MOREX.r3.5HG0517490 | Protein kinase | Looseley et al. (2012) |
| *qSc.APR25* | JHI-Hv50k-2016-358258 | 5 | 582.5315 | HORVU.MOREX.r3.5HG0530180 | Kelch repeat-containing family protein | Daba et al. (2019), |
| *qSc.APR26* | JHI-Hv50k-2016-367980 | 6 | 4.3492 | HORVU.MOREX.r3.6HG0540120 | Senescence-associated protein DIN1 | Jensen et al. (2002), Cheong et al. (2006) |
| *qSc.APR27* | JHI-Hv50k-2016-446947 | 7 | 10.7460 | HORVU.MOREX.r3.7HG0640950 | Fe superoxide dismutase 2 | Schweizer et al. (1995) |
| *qSc.APR28* | JHI-Hv50k-2016-516264 | 7 | 627.5568 | HORVU.MOREX.r3.7HG0748760 | GDSL esterase/lipase | Cheong et al. (2006), Genger et al. (2005) |
